# Transcriptional changes of proteins of the thioredoxin and glutathione systems in *Acanthamoeba* spp. under oxidative stress – an RNA approach

**DOI:** 10.1051/parasite/2022025

**Published:** 2022-05-09

**Authors:** Martina Köhsler, David Leitsch, Alvie Loufouma Mbouaka, Maximilian Wekerle, Julia Walochnik

**Affiliations:** 1 Institute of Specific Prophylaxis and Tropical Medicine, Center for Pathophysiology, Infectiology and Immunology, Medical University of Vienna 1090 Vienna Austria

**Keywords:** *Acanthamoeba castellanii*, Oxidative stress, Thioredoxin system, Glutathione system, Auranofin

## Abstract

The thioredoxin (Trx) and the glutathione (GSH) systems represent important antioxidant systems in cells and in particular thioredoxin reductase (TrxR) has been shown to constitute a promising drug target in parasites. For the facultative protozoal pathogen *Acanthamoeba*, it was demonstrated that a bacterial TrxR as well as a TrxR, characteristic of higher eukaryotes, mammals and humans is expressed on the protein level. However, only bacterial TrxR is strongly induced by oxidative stress in *Acanthamoeba castellanii*. In this study, the impact of oxidative stress on key enzymes involved in the thioredoxin and the glutathione system of *A. castellanii* under different culture conditions and of clinical *Acanthamoeba* isolates was evaluated on the RNA level employing RT-qPCR. Additionally, the effect of auranofin, a thioredoxin reductase inhibitor, already established as a potential drug in other parasites, on target enzymes in *A. castellanii* was investigated. Oxidative stress induced by hydrogen peroxide led to significant stimulation of bacterial TrxR and thioredoxin, while diamide had a strong impact on all investigated enzymes. Different strains displayed distinct transcriptional responses, rather correlating to sensitivity against the respective stressor than to respective pathogenic potential. Culture conditions appear to have a major effect on transcriptional changes in *A. castellanii*. Treatment with auranofin led to transcriptional activation of the GSH system, indicating its role as a potential backup for the Trx system. Altogether, our data provide more profound insights into the complex redox system of *Acanthamoeba*, preparing the ground for further investigations on this topic.

## Abstract (fr)

Les systèmes de la thiorédoxine (Trx) et du glutathion (GSH) représentent des systèmes antioxydants importants dans les cellules et, en particulier, la thiorédoxine réductase (TrxR) s’est avérée constituer une cible médicamenteuse prometteuse chez les parasites. Pour le pathogène protozoaire facultatif *Acanthamoeba*, il a été démontré qu’une TrxR bactérienne ainsi qu’une TrxR, caractéristique des eucaryotes supérieurs, des mammifères et des humains, s’expriment au niveau protéique. Cependant, seule la TrxR bactérienne est fortement induite par le stress oxydatif chez *Acanthamoeba castellanii*. Dans cette étude, l’impact du stress oxydatif sur les enzymes clés impliquées dans la thiorédoxine et le système glutathion d’*A. castellanii* dans différentes conditions de culture et d’isolats cliniques d’*Acanthamoeba* a été évalué au niveau de l’ARN en utilisant la RT-qPCR. De plus, l’effet de l’auranofine, un inhibiteur de la thiorédoxine réductase déjà établi comme médicament potentiel chez d’autres parasites, a été étudié sur les enzymes cibles chez *A. castellanii*. Le stress oxydatif induit par le peroxyde d’hydrogène a conduit à une stimulation significative du TrxR bactérien et de la thiorédoxine tandis que le diamide a eu un fort impact sur toutes les enzymes étudiées. Différentes souches ont affiché des réponses transcriptionnelles distinctes, plutôt corrélées à la sensibilité contre le facteur de stress respectif qu’à leur potentiel pathogène respectif. Les conditions de culture semblent avoir un effet majeur sur les changements transcriptionnels chez *A. castellanii*. Le traitement à l’auranofine a conduit à une activation transcriptionnelle du système GSH, indiquant son rôle de sauvegarde potentielle pour le système Trx. Dans l’ensemble, nos données fournissent des informations plus approfondies sur le système redox complexe d’*Acanthamoeba*, préparant le terrain pour de nouvelles investigations sur ce sujet.

## Introduction

Free-living amoebae of the genus *Acanthamoeba* are bacteriovores ubiquitously spread in the environment, but they also have pathogenic potential. *Acanthamoeba* spp. cause sight-threatening *Acanthamoeba* keratitis (AK) mostly occurring in contact lens wearers, or act as opportunistic pathogens in immunocompromised individuals causing granulomatous amoebic encephalitis (GAE) [26]. Both disease patterns are difficult to treat due to the lack of specific therapeutic agents. While in AK, this can lead to recurring infections and eventually loss of vision, GAE more dramatically constitutes a fatal disease in almost all cases [23].

Recent insights into the genome of *Acanthamoeba castellanii* revealed that these organisms possess unique metabolic strategies and a highly evolved cellular repertoire, most likely arisen from extensive lateral gene transfer [5].

With access to the genome, it could be established that acanthamoebae possess all components of a thioredoxin (Trx) and a glutathione (GSH) system. Both networks harness NADPH to reduce disulfide bonds, thus keeping the cellular environment in a reduced state. The Trx system is composed of peroxiredoxins (Prx), thioredoxin (Trx) and thioredoxin reductase (TrxR), while the GSH system is composed of glutaredoxin (Grx), glutathione, glutathione reductase (GR) and glutathione peroxidase (Gpx) [38]. Thioredoxins and glutaredoxins are structurally similar and display considerable functional overlap being hydrogen donors for a number of metabolic enzymes. However, while thioredoxin is reduced by thioredoxin reductase, glutaredoxin is reduced by glutathione, which in return is reduced by glutathione reductase. Both systems have important functions in the cell, such as DNA synthesis, protein folding or cell signaling and are indispensable for cellular antioxidant defense, by managing reactive oxygen species (ROS) and by repairing oxidative damage [18, 28].

Acanthamoebae have a distinctive Trx system, which comprises two different TrxRs. While in most other organisms either a high-molecular weight TrxR (TrxR-L) or a low-molecular weight TrxR (TrxR-S) is present, acanthamoebae possess genes coding for both types. TrxR-L is typically found in higher eukaryotes, mammals and humans, while TrxR-S is usually found in bacteria, fungi, plants and lower eukaryotes. In a previous study, we already established that both TrxRs in *A. castellanii* strain Neff are expressed on the protein level and localized in the cytosol. Interestingly, however, only TrxR-S was shown to be upregulated upon oxidative stress on the mRNA and protein level [22].

The Trx system has also been shown to be a potential drug target in various pathogens. Auranofin, a gold-based compound that inhibits TrxR activity, has already been established as a promising drug candidate with robust activity against several protozoan parasites, including *Entamoeba histolytica* [9], *Plasmodium falciparum* [37] or *Giardia* spp. [39]. Potent efficacy has also been demonstrated against *Acanthamoeba* spp. **[** 24, 36 **]**.

In this study, transcriptional changes of key enzymes (TrxR-L, TrxR-S, Trx, Prx, Grx, GR, Gpx) of the Trx and GSH system of *A. castellanii* strain Neff and three clinical *Acanthamoeba* isolates in response to oxidative stress were evaluated in detail, in order to assess whether pathogenic potential is reflected by the respective transcription profile. Additionally, in strain Neff, the effect of different culture conditions and incubation with auranofin on the RNA expression of these enzymes was evaluated.

## Material and methods

### Strains and growth conditions

Four *Acanthamoeba* strains, all representing genotype T4, were used in this study: *A. castellanii* strain Neff (ATCC^®^ 30010™), isolated from soil in the year 1957, and three strains isolated from corneal scrapings of patients, who had developed *Acanthamoeba* keratitis, namely strain 2HH, isolated in the year 2000, strain WIR17, isolated in 2017, and strain JEA19, isolated in 2019. All strains were grown at 25 °C in 20 mL sterile filtrated proteose peptone yeast extract-glucose medium (PYG) in 75-cm^2^ tissue culture flasks with weekly medium changes and regular sub-culturing.

### Treatment of strains with hydrogen peroxide, diamide and auranofin

3 × 10^6^ amoebae of strain Neff were counted using a Fuchs-Rosenthal hemocytometer, transferred to 10 mL fresh PYG and exposed to 750 μM hydrogen peroxide (H_2_O_2_) and 2 mM diamide for two and six hours, respectively. For treatment with auranofin, 3 × 10^6^ amoebae were grown in the presence of 10 μM and 20 μM auranofin for 48 h. In all experiments, amoebae in PYG were used as controls.

For Neff plate cultures (NeffPl), 2 × 10^5^ amoebae from liquid culture were transferred onto NN-agar plates coated with *E. coli* and grown for 72 h. Subsequently, amoebae were harvested and then exposed to 750 μM H_2_O_2_ and 2 mM diamide for two hours in PBS. Untreated amoebae were used as a control.

Strains 2HH, WIR17 and JEA19 were cultured under the same conditions as strain Neff and exposed to 750 μM H_2_O_2_ and 2 mM diamide for two hours, as described for strain Neff with amoebae in PYG as controls.

### RNA isolation and cDNA synthesis

For all preparations, amoebae were counted using a Fuchs-Rosenthal hemocytometer prior to exposure to stress conditions. 3 × 10^6^ amoebae were harvested by centrifugation (700 ×*g*/10 min) and washed with PBS. RNA was isolated using a GeneJET RNA Purification Kit (Thermo Fisher Scientific), following the manufacturer’s instructions. RNA was treated with DNase I (Roche) to remove contaminating genomic DNA. The RNA concentration and purity were determined using a NanoDrop spectrophotometer ND1000 (NanoDrop Technologies). Only samples with a 260/280 ratio between 1.8 and 2.0 were used for subsequent analyses. RNA integrity was assessed by 1% agarose gel electrophoresis. For cDNA synthesis, the amount of total RNA was standardized to 1 μg per reaction. First-strand cDNA was synthesized using the Maxima First Strand cDNA Synthesis Kit for RT-qPCR (Thermo Fisher Scientific). Then, cDNA was quantified using the ND1000. All cDNA samples were diluted to 10 ng/μL using DEPC-treated water and used immediately after cDNA synthesis or stored at −80 °C for further processing.

### Quantitative real-time PCR

RT-qPCRs targeting TrxR of the low-molecular-weight type (TrxR-S), TrxR of the high-molecular-weight type (TrxR-L), thioredoxin-1 (Trx-1), two peroxiredoxins (Prx-2, Prx-3), two glutaredoxins (Glx-1, Glx-2), glutathione-disulfide reductase (GR) and glutathione peroxidase (Gpx) were performed as described previously [22]. Information on primers used is provided in Table 1. In brief, RT-qPCRs were carried out in a CFX96 thermocycler (Bio-Rad, Vienna, Austria) using Takyon No Rox SYBR Master Mix dTTP Blue (Eurogentec). The RT-qPCR temperature profile included an initial denaturation step at 95 °C for 3 min, followed by 45 cycles of 15 s at 95 °C, 15 s at 55 °C and 15 s at 72 °C. Experiments were carried out in three independent set ups (strain Neff H_2_O_2_, diamide, auranofin) or in two independent set ups (Neff plate, 2HH, WIR17 and JEA9). For each cDNA batch, three independent RT-qPCR runs were performed. The relative expression of target genes was normalized towards the 18S rRNA-gene, employing primers designed by Qvarnstrom et al. [34] and the hypoxanthine-guanine phosphoribosyltransferase (HPRT) employing the 2^−ΔΔ*Ct*^ method [33]. Experiments with H_2_O_2_ were only normalized towards the 18S-rRNA-gene, since H_2_O_2_ affected the HPRT of some strains and led to a biased output, impeding a direct comparison of different strains.

**Table 1:** Primers used for RT-qPCR in this study.

Target	Source (Genbank)	Target gene	Primer sequence (5′ – 3′)	Amplicon length (bp)	Average Tm (°C)	Amplification efficiency (%)
TrxR-L_fw	XM_004353581	thioredoxin reductase 1	GGATTCGACCAACAGCTCG	212	59.5	96.3
TrxR-L_rev		cytoplasmic, putative	GGATTCGACCAACAGCTCG		59.5	
TrxR-S_fw	M_004351629	thioredoxin-disulfide	CTCTCGAACCCCAAGATC	182	56.3	97.3
TrxR-S_rev		reductase	CACCTGACCATTCAGGAAC		57.5	
Trx-1_fw	XM_004335461	thioredoxin-1, putative	GGACTTCTTTGCCACGTG	184	56.3	95.5
Trx-1_rev			GCACCTTGCTGCCGTTC		57.3	
Prx-2_fw	XM_004333592	peroxiredoxin 2	GACACACCTACCGTGGTC	226	58.4	77.9
Prx-2_rev			GTTGACCTTCTCGAAGTAGG		58.4	
Prx-3_fw	XM_004348492	2cys peroxiredoxin	CAACGACTTGCCAGTGGG	157	58.4	94.1
Prx-3_rev			CCTTGAAGTACTCCTTGGAC		58.4	
Grx-1_fw	XM_004339700	glutaredoxin, putative	CGCCAAGAACACCGTTATG	203	57.5	90.3
Grx-1_rev			CCAATGTGCTCACTGTCGATG		56.3	
Grx-2_fw	XM_004339719	glutaredoxin, putative	GGAGATGAGAGCGTTCAG	184	56.3	98.8
Grx-2_rev			CTCTTGGCCTGCATCTCG		58.4	
GR_fw	XM_004338198	glutathione-disulfide	CGACACTCTCTACAACAACC	149	59.5	90.6
GR_rev		reductase	CTTCTCGTCACGCTTGGAC		61.6	
Gpx_fw	XM_004335951	glutathione peroxidase Hyr1	CTGCAACCAGTTCGGCAG	193	58.4	91.7
Gpx_rev			CGAAGTTCCACTTGATGCG		57.5	
18S_fw		18S-rRNA-gene	CCCAGATCGTTTACCGTGAA	180	58.4	97.5
18S_rev			TAAATATTAATGCCCCCAACTATCC		60.9	
HPRT_fw	XM_004337011	hypoxanthine-guanine phosphoribosyltransferase	GGAGCGGATCGTTCTCTG	201	58.4	102
HPRT_rev			ATCTTGGCGTCGACGTGC		58.4	

### Resistance of strains to H_2_O_2_ and diamide

To evaluate the differences in susceptibility to higher concentrations of H_2_O_2_ and diamide, 2 × 10^5^ amoebae of all strains were harvested and resuspended in sterile PBS and exposed to 1.5 mM H_2_O_2_ and 5 mM diamide for two hours. The experiments were performed in 16-well plates. Following incubation, amoeba viability was determined by adding 0.1% trypan blue and evaluating the number of live (non-stained) and dead (stained) amoebae using a Fuchs-Rosenthal hemocytometer. Amoebae with PBS alone were used as controls. The number of stained cells was subtracted from the total, indicating the death count. Data are represented as the means and standard deviation of the mean (SEM) of at least three independent experiments performed in duplicate.

### Statistics

Statistical significance of the data was determined using multiple comparisons with a Kruskal–Wallis test on ranks followed by a Dunn’s post hoc test and/or a non-parametric Mann–Whitney *U* test (GraphPad 9 software). For normalization with both reference genes, *p*-values were determined for both normalizations and only those values that were statistically significant using both normalizations were considered as truly significant. *p* < 0.05 was considered significant.

## Results

### Effect of oxidative stress on target genes in *A. castellanii* stain Neff after two and six hours

**Figure 1:**
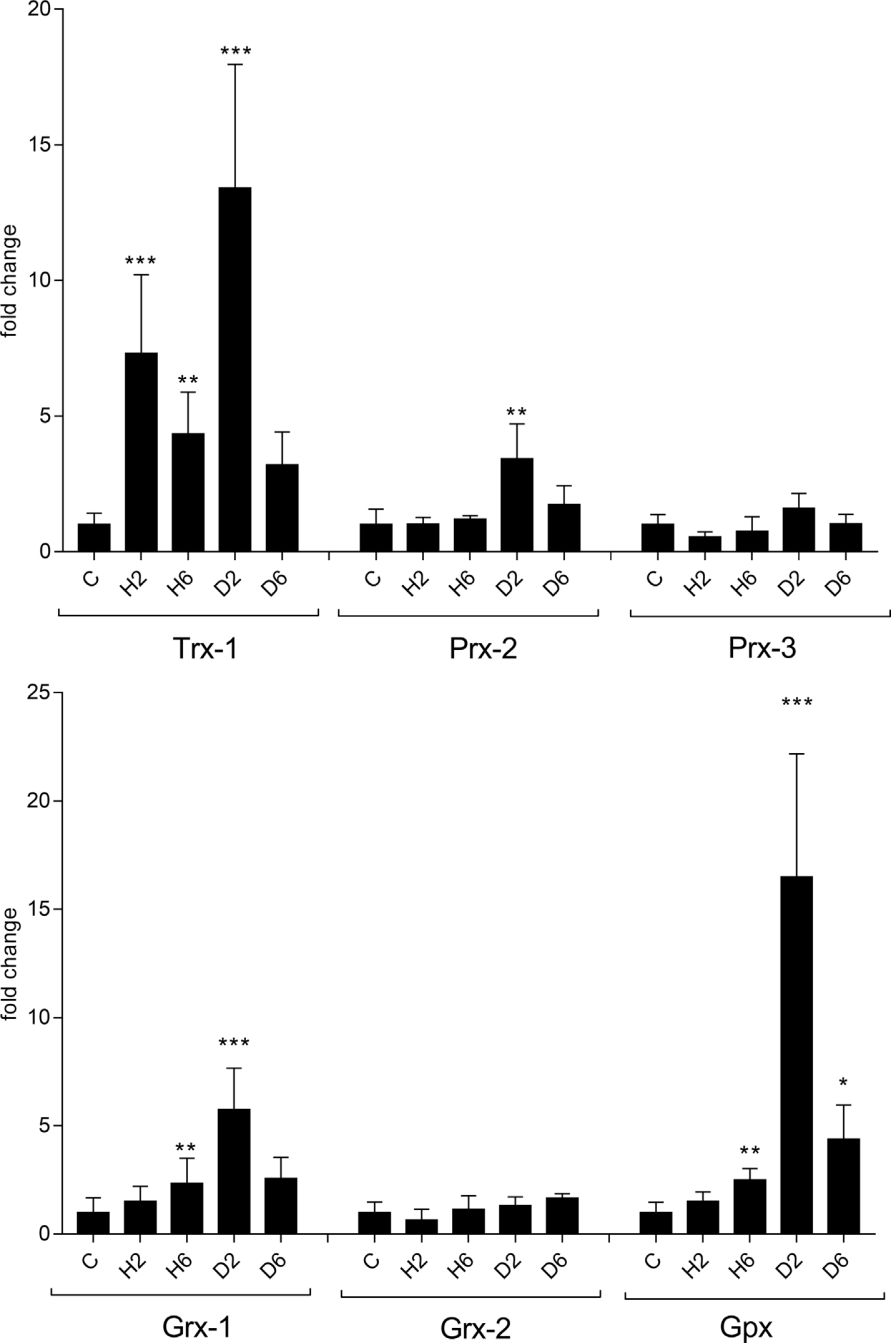
RNA expression of investigated target genes in *A. castellanii* strain Neff after challenge with 750 μM H_2_O_2_ for two (H2) and six (H6) hours and 2 mM diamide for two (D2) and six (D6) hours. (a) Thioredoxin 1 (Trx-1), peroxiredoxin 2 (Prx-2), peroxiredoxin 3 (Prx-3). (b) Glutaredoxin 1 (Grx-1), glutaredoxin 2 (Grx-2), glutathione peroxidase (Gpx); C: untreated control. The *y*-axis indicates – fold increase of RNA levels as compared to untreated controls. Error bars show the standard deviation of the mean (SEM). All values were obtained from at least three biological replicates in triplicate. *p < 0.05, **p < 0.01, and ***p < 0.0001 according to statistical analysis (multiple comparisons with a Kruskal–Wallis test on ranks followed by Dunn’s post hoc test).

Fold changes for RNA expression after exposure to H_2_O_2_ and diamide after two and six hours, respectively, are shown in Figure 1. Exact numbers for fold changes (FC) and standard deviations (STD) for all experiments are provided in Supplementary Table 1.

The most pronounced effect after exposure to H_2_O_2_ was obseverd for Trx-1 with fold changes of 7.3 and 4.3 after two and six hours, respectively. Interestingly, both peroxiredoxins (Prx-2 and Prx-3) exhibited no significant increase of RNA expression in response to H_2_O_2_. Prx-3 expression actually decreased by almost 50%. Expression of Grx-1 was slightly elevated, while Grx-2 was reduced. Surprisingly, RNA expression of Gpx was also only minimally increased after two hours.

Diamide had a more pronounced effect on the expression of most target genes, with Trx-1 and Gpx exhibiting highly significant fold changes after two hours of 13.4 and 18.3, respectivley, but also Prx-2 and Grx-1 showed notable changes in RNA expression. Expression of Prx-3 and Grx-2 on the other hand appeared to be unaffected by diamide. After six hours of exposure to diamide, fold changes generally decreased, indicating that the effect of diamide is most pronounced within the first few hours of exposure.

### Effect of oxidative stress on target genes in *A. castellanii* stain Neff after plate culture and pathogenic *Acanthamoeba* isolates after two hours

**Figure 2:**
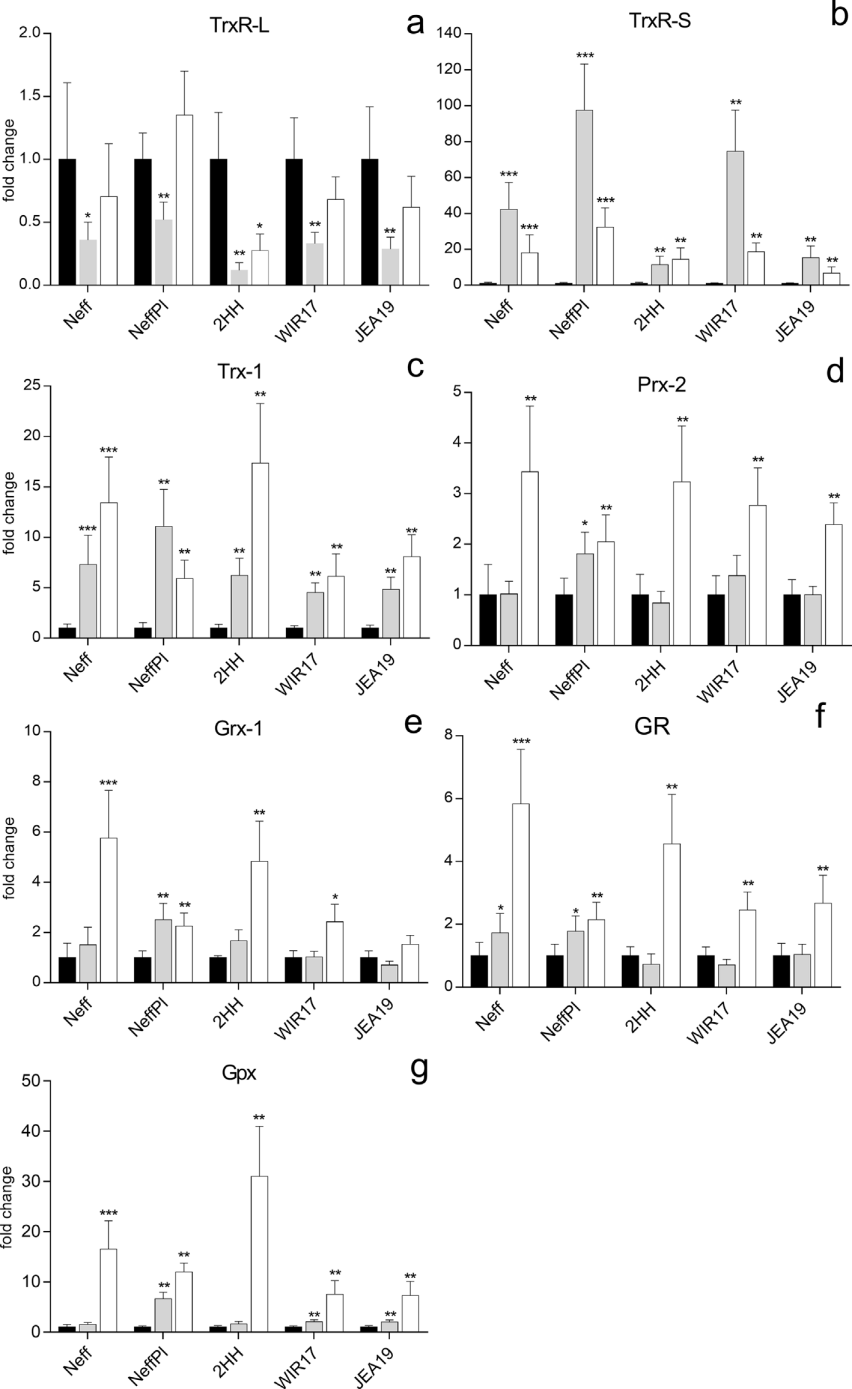
RNA expression of investigated target genes in strain Neff, strain Neff after plate culture (NeffPl), and three clinical *Acanthamoeba* isolates (2HH, WIR17, JEA19) after challenge with H_2_O_2_ and diamide for two hours. (a) High-molecular weight thioredoxin reductase (TrxR-L), (b) low-molecular weight thioredoxin reductase (TrxR-S), (c) thioredoxin 1 (Trx-1), (d) peroxiredoxin 2 (Prx-2), (e) glutaredoxin 1 (Grx-1), (f) glutathione reductase (GR), (g) glutathione peroxidase (Gpx). Black bars: untreated control; grey bars: treated with 750 μM H_2_O_2_; white bars: treated with 2 mM diamide. The *y*-axis indicates – fold increase of mRNA levels as compared to untreated controls. Error bars show the standard deviation of the mean (SEM). Values were obtained from at least two biological replicates in triplicate. *p < 0.05, **p < 0.01, and ***p < 0.0001 according to statistical analysis (non-parametric Mann–Whitney *U* test).

Fold changes for all investigated target genes for strain Neff, strain Neff plate cultures (NeffPl) and three clinical isolates (2HH, WIR17, JEA19) are shown in Figure 2, with exact numbers for fold changes (FC) and standard deviations (STD) provided in Supplementary Table 2.

Fold changes for strains Neff TrxR-L, TrxR-S and GR were already published in Leitsch et al. [22], but were included in this figure for comparison with investigated clinical isolates in this study.

TrxR-L fold changes of all strains were quite similar, with strong reduction of RNA expression when exposed to H_2_O_2_ ranging from approximately 50% (NeffPl) to 90% reduction (2HH). The decrease in RNA expression was less pronounced after treatment with diamide. Only in NeffPl, diamide led to a slight increase in RNA expression (Fig. 2a).

As expected, TrxR-S expression was significantly enhanced both after exposure to H_2_O_2_ and to diamide in all strains; however, the differences in respective fold changes were considerable (Fig. 2b). As already reported by Leitsch et al. [22], in strain Neff TrxR-S expression increases approximately by a factor 50, when exposed to H_2_O_2_. Obviously even a short time under different culture conditions, in this case on agar plates coated with *E. coli*, has a strong impact on the TrxR-S response to H_2_O_2_, since NeffPl exhibited an increase in TrxR-S expression almost twice as high (fold change 97.5). Also, in strain WIR17, the fold change of TrxR-S was significant with 74.7-fold. Strains 2HH and JEA19 showed a similar expression pattern; however, fold changes for these strains were considerably lower with 11.3 and 15, respectively. Differences in TrxR-S expression after exposure to diamide showed a similar pattern with NeffPl exhibiting the highest fold change (32.3), followed by strain Neff, WIR17 and 2HH, while strains JEA19 exhibited the lowest fold change of 6-fold. On Trx-1 transcription, both H_2_O_2_ and diamide had a pronounced effect in all strains. Trx-1fold changes ranged from 4.5 (WIR17) to 11.1 (NeffPl) after H_2_O_2_ exposure and from 5.9 (NeffPl) to 15.1 (2HH) after diamide exposure (Fig. 2c). Prx-2 expression after H_2_O_2_ exposure was unaltered in all strains, while diamide led to a moderate increase in all strains ranging from 2.1 for strain 2HH to 3.4 for strain Neff (Fig. 2d). Grx-1 expression was not affected by treatment with H_2_O_2_ in clinical *Acanthamoeba* strains, but had some effect on expression in strain Neff for both culture conditions. More differences could be observed for treatment with diamide with relatively high fold changes, of around 6 for strains Neff and 2HH, and less pronounced increases in expression for strains NeffPl, WIR17 and JEA19 (Fig. 2e). The situation was rather similar for GR with strains Neff and 2HH exhibiting more pronounced fold changes and generally lower fold changes in other strains (Fig. 2f). As already presented for strain Neff, interestingly, H_2_O_2_ had only minimal effects on the RNA expression of Gpx, while diamide exposure led to significant fold changes in all strains, although considerable differences could be observed. While strain 2HH exhibited a fold change of almost 28 and strain Neff of 18.2, for strain JEA19 Gpx transcription only increased 5.7-fold. However, this is still noteworthy compared to all other target genes studied (Fig. 2g).

### Sensitivity to elevated concentrations of H_2_O_2_ and diamide

In this experiment, higher, potentially lethal concentrations of H_2_O_2_ and diamide were applied, in order to verify a difference in susceptibility of the investigated strains.

**Figure 3:**
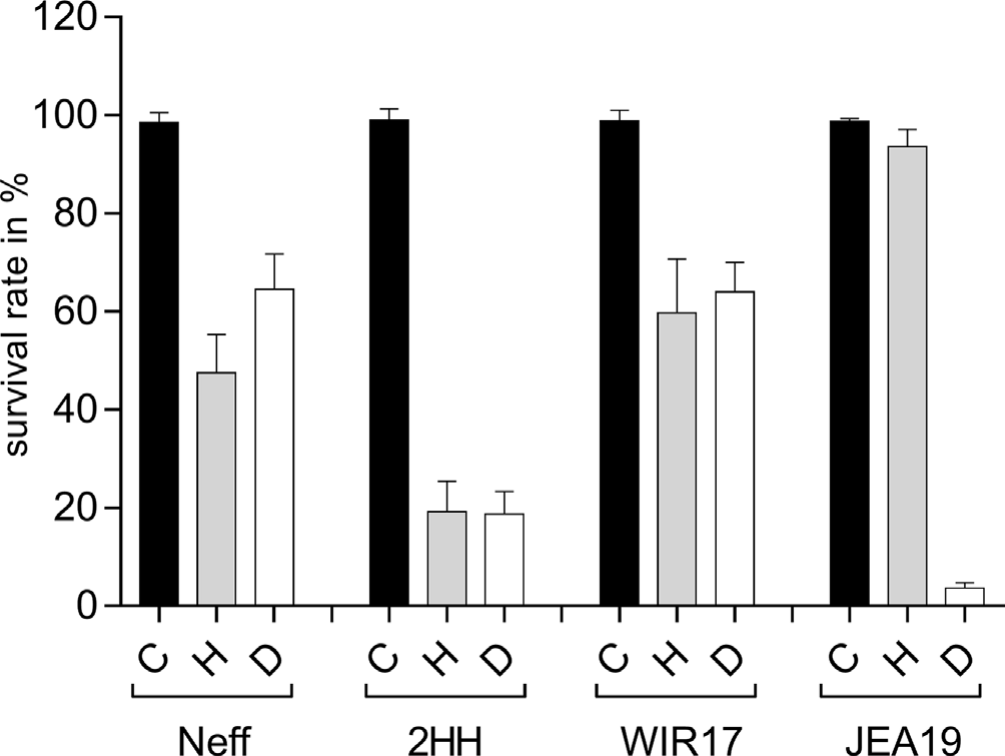
Survival rates of investigated *Acanthamoeba* strains after challenge with elevated concentrations of H_2_O_2_ and diamide for two hours. C: untreated control; H: treated with 1.5 mM H_2_O_2_; D: treated with 5 mM diamide. Values were obtained from three biological replicates in triplicate. Error bars show the standard deviation of the mean (SEM).

Interestingly, considerable differences in susceptibility to H_2_O_2_ could be observed (Fig. 3). While strain JEA19 was highly resistant against 1.5 mM H_2_O_2_ with almost all amoebae surviving incubation for two hours (survival rate 95%), in strain 2HH, this concentration led to a substantial reduction of living cells with survival rates of only 20%. Strains Neff and WIR17 showed moderate susceptibility, with survival rates of 48% and 60%, respectively.

Also, susceptibility to diamide varied significantly between strains. Here, however, strain JEA19, which was unaffected by H_2_O_2_, was highly sensitive with only 3% survival rate after exposure to 5 mM diamide. Strain 2HH was also highly susceptible with 19% survival rate, while strains Neff and WIR17 showed similar, quite moderate, responses with survival rates of 65% and 60%, respectively.

### Effect of auranofin on expression of target genes in *A. castellanii* strain Neff

**Figure 4:**
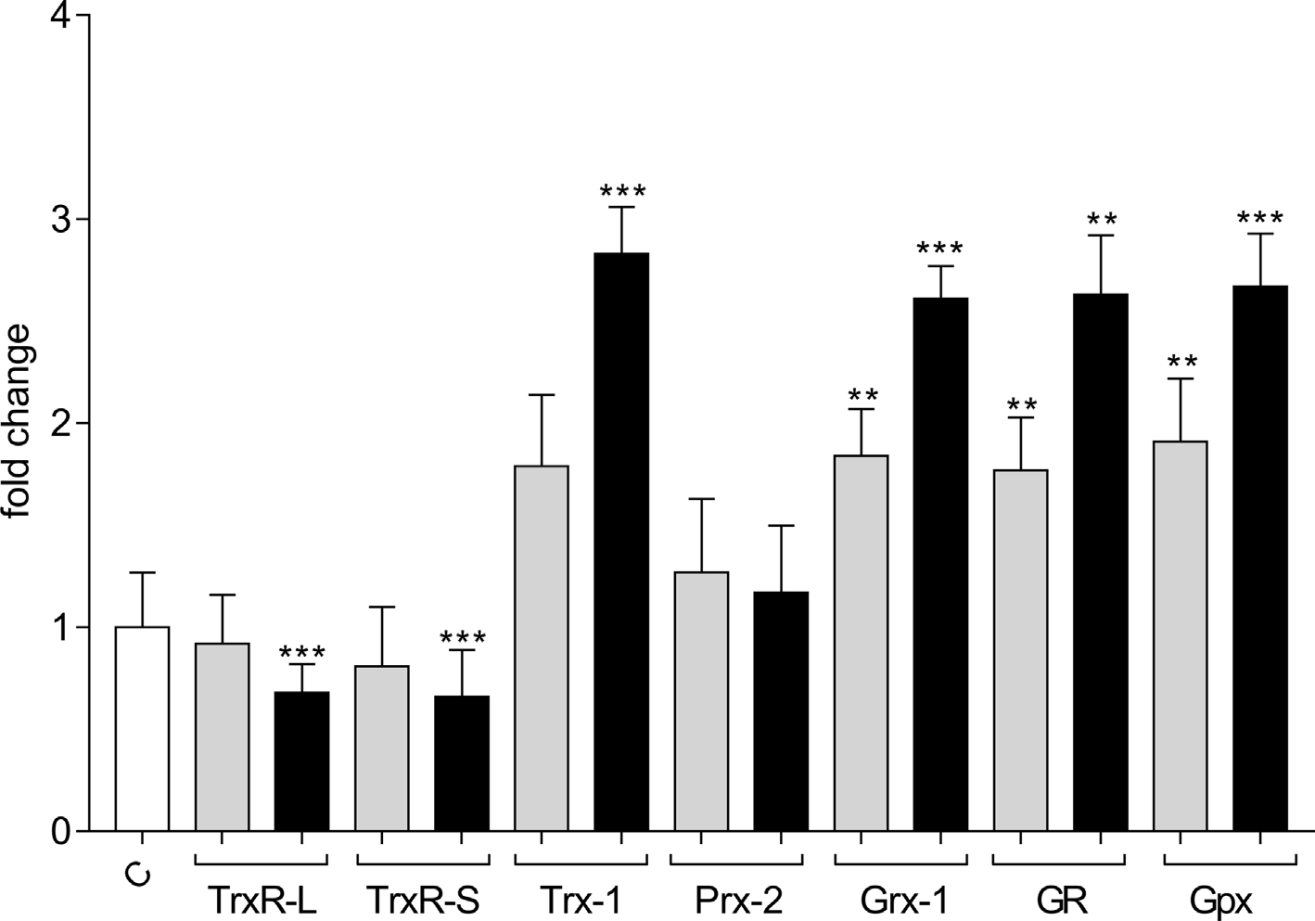
RNA expression of investigated target genes in strain Neff after treatment with auranofin for 48 h. High-molecular weight thioredoxin reductase (TrxR-L), low-molecular weight thioredoxin reductase (TrxR-S), thioredoxin 1 (Trx-1), peroxiredoxin 2 (Prx-2), glutaredoxin 1 (Grx-1), glutathione reductase (GR), glutathione peroxidase (Gpx), untreated control (C). Grey bars: 10 μM auranofin; black bars: 20 μM auranofin. The y-axis indicates – fold increase of mRNA levels as compared to untreated controls. Error bars show the standard deviation of the mean (SEM). Values were obtained from at least three biological replicates in triplicate. *p < 0.05, **p < 0.01, and ***p < 0.0001 according to statistical analysis (nonparametric Mann–Whitney *U* test).

Treatment with 10 μm and 20 μm auranofin for 48 h in strain Neff resulted in a reduction of RNA expression of TrxR-L and TrxR-S in a dose-dependent manner. The decrease in RNA expression was more pronounced for TrxR-S, with a reduction of 40%. All other investigated target genes, apart from Prx-2 showed a dose dependent, significant increase of RNA expression between two and three-fold (Fig. 4, Supplementary Table 2).

## Discussion

The fact that *Acanthamoeba* possesses two different types of TrxR is quite intriguing, since only very few organisms were reported so far with this feature, for instance *Naegleria fowleri* [6]. While our primary focus lay on the TrxRs and GR in *Acanthamoeba castellanii* strain Neff [22], in this study we attempted a more detailed evaluation of other enzymes involved in the redox systems of acanthamoebae. Moreover, the expression patterns of clinical strains were investigated in order to evaluate whether pathogenic strains exhibit a distinctive response to oxidative stress.

Similar to the experiments already published on the TrxRs and GR [22] in strain Neff, treatment with H_2_O_2_ and diamide was investigated after two and six hours, with most pronounced RNA expression changes after two hours, obviously regressing to initial levels after that, indicating that most transcriptional changes occur earlier after oxidative challenge with H_2_O_2_ and diamide. The most pronounced increases in RNA expression could be observed for Trx-1 and Gpx after treatment with diamide for two hours. Since changes in Prx-3 and Grx-2 transcription were only detected for H_2_O_2_ treatment after two hours with decreasing RNA levels, these two enzymes were considered to be not involved in oxidative defense and not further investigated in all other experiments. Furthermore, it cannot be ruled out that Grx-2 (XM_004339719) might be incorrectly annotated. When searching for homologous sequences employing the BLAST application, the sequence of Grx-2 does not yield any results, while the sequences of all other investigated targets show homologous counterparts in other organisms.

Similar to observations for strain Neff, treatment of other strains with H_2_O_2_ and diamide resulted in a strong increase of TrxR-S transcription, while TrxR-L transcription was down-regulated. Increase of TrxR-S transcription was typically more pronounced after challenge with H_2_O_2_; interestingly, Trx-1 transcription was more affected by diamide in most strains. Also in *Listeria monocytogenes*, where thioredoxin A was shown to be closely linked to pathogenicity, diamide was identified as a stronger inducer of thioredoxin than H_2_O_2_, indicating that different stressors result in distinct expression patterns of Trx and other genes involved in oxidative stress response [4]. An explanation why TrxR-S expression does not necessarily correlate with Trx-1 expression in our study might be that TrxRs do not only participate in the reduction of thioredoxin, but have a wider substrate range [21]. In the fungus *Alternaria alternata*, it was shown that both TrxR and GR play an important role in cellular resistance to oxidative stress; however, here exposure to H_2_O_2_ resulted in pronounced induction of GR expression, while only moderate changes in TrxR expression were reported [25]. In our study, however, GR expression was only minimally affected by H_2_O_2_, while the observed fold changes for TrxR-S are comparable to fold changes for GR in *Alternaria alternata*. In contrast to these observations are findings in *Candida albicans*, where H_2_O_2_ resulted in similar RNA expression changes for Trx, TrxR and GR; however, for all three targets only to a 4-fold extent [11]. An important role for TrxR in resistance against H_2_O_2_ has also been reported for *Dictyostelium discoideum*, where H_2_O_2_ led to strong induction of TrxR, while cells with TrxR under-expression were highly sensitive to H_2_O_2_. Additionally, in *D. discoideum*, TrxR appears to be involved in cell growth [20].

Generally, diamide led to stronger induction of components of the GSH system, while H_2_O_2_ had no or only minor effects. The difference between expression profiles in response to H_2_O_2_ and diamide might be explained by a different mode of activation of transcription, which has been demonstrated for yeast [32]. H_2_O_2_ and thiol-reactive chemicals activate Yap1, the transcription factor mediating adaptive responses to oxidative stress in *Saccharomyces cerevisiae* differently, resulting in a distinct expression of protective genes. Furthermore, it was shown that diamide was the most pleiotropic stress-inducing agent, not only inducing transcription of genes involved in oxidative stress response, but also in protein folding and respiration in yeast [15]. That might explain the more pronounced effect of diamide on most investigated target genes in our study. Also in *Cryptococcus neoformans,* where TrxR has been shown to be essential for viability, oxidative stress induced by H_2_O_2_ led to a much weaker increase in RNA expression than nitric oxide stress [29].

Culture conditions obviously have a considerable influence on the response to oxidative stress in strain Neff, although amoebae were transferred onto agar plates only a few days prior to RNA isolation. H_2_O_2_ had a significantly stronger effect on RNA expression of plate cultures, while diamide obviously leads to more pronounced transcriptional changes in axenic cultures. For both stressors, however, TrxR-S transcription was twice as high compared to axenic cultures. Discrepancies might be explained by the different amounts of oxygen in liquid and plate culture, but also the respective growth phases might be responsible for varying expression rates. In liquid culture, nutrients are more or less omnipresent, while they are limited on agar plates. Therefore, amoebae on agar plates might reach the stationary phase earlier, while amoebae in axenic culture pursue exponential growth for a longer period of time. Differences in response to H_2_O_2_ during different growth phases have already been observed for *A. castellanii* investigating mitochondrial function under oxidative stress [41]. Also in yeast, it was shown that thioredoxin is particularly required for oxidative stress situations in the stationary growth phase [14]. In *C. albicans*, growth phase specific transcriptional profiles accompanied by different resistances to oxidative stress were reported, with the nutritional state also suggested as a redox state influencing factor [27].

An unexpected finding was that diamide but not H_2_O_2_ resulted in a significant induction of Gpx transcription, since Gpxs are generally considered to catalyze the reduction of H_2_O_2_ to H_2_O, by oxidizing reduced glutathione during oxidative stress [3, 35]. An explanation might be that components of the Trx system are sufficient in combating oxidative stress due to H_2_O_2_, as indicated by the strong induction of TrxR-S and Trx-1. The considerable increase of Gpx expression after diamide treatment (Figs. 1 and 2), however, might be due to other functions of Gpx, since in addition to the removal of H_2_O_2_, another key function of Gpxs is the reduction of lipid hydroperoxides [13]. Diamide, a potent thiol-oxidizing agent, accomplishes fast oxidation of GSH to GSSG, resulting in a depletion of reduced glutathione in the cell. This might facilitate lipid peroxidation, as described previously [2]. The need to reduce lipid peroxides might contribute to the strong increase in Gpx transcription. Furthermore, the Gpx of *Acanthamoeba* appears to be a seleno-independent Gpx, since it does not contain a UGA codon [22]. For seleno-independent Gpxs, various other functions in addition to their scavenging properties have been proposed [17].

In general, transcription patterns of different *Acanthamoeba* strains investigated were similar. However, the magnitude of RNA increase of the investigated target genes varied to a great extent between strains. This was particularly pronounced for TrxR-S and Gpx transcription. The concentrations applied for H_2_O_2_ and diamide challenges were tested in advance and were well tolerated by all stains. The chosen concentrations were supposed to induce oxidative stress without harming the cell irreversibly in order to obtain comparable results. When we discovered that transcriptional responses of the investigated strains were quite divergent, we tested more challenging concentrations to evaluate potential differences in sensitivity to H_2_O_2_ and diamide, which were shown to be more substantial than expected. While strains Neff and WIR17 exhibited similar resistances against both stressors, strain 2HH was shown to be very sensitive to higher concentrations of both agents. Interestingly, strain JEA19 was almost unaffected by H_2_O_2_ but the most sensitive strain in response to treatment with diamide. It would be conceivable that a strain struggling to survive reacts with more different strategies than a strain only minimally affected. In *Schizosaccharomyces pombe*, it was shown that depending on the level of H_2_O_2_, different transcriptional pathways are activated, either facilitating adaptation or survival as a response [40]. Presumably, there is a unique response for each strain depending on its specific characteristics. The pathogenic potential of a strain, however, was not reflected by a specific expression pattern of the investigated strains. Also, the different activity of superoxide dismutase and catalase, other potential antagonists against oxidative stress, might contribute to variable responses and susceptibilities of strains. An induction of these enzymes in response to treatment with H_2_O_2_ and potential protection against apoptosis has just recently been demonstrated for *Acanthamoeba* sp. [30].

Another aim of this study was to obtain more data on the action of peroxiredoxins. Prxs specifically decompose several kinds of hydroperoxides, including H_2_O_2_, by providing reducing equivalents. In return, thioredoxin serves as an electron donor for Prxs [1, 8, 35]. In *Acanthamoeba*, four Prxs were identified in the genome [22]. Unfortunately, only for two of these Prxs (Prx-2 and Prx-3), a properly functioning protocol could be established. These two Prxs, however, appear to play a subordinate role in defense against oxidative stress, since treatment with H_2_O_2_ did not lead to any significant transcriptional changes. These changes might occur earlier after treatment, since hydrogen peroxide scavenging by Prxs is a fast process. Alternatively, recycling by the Trx system combined with the high abundance of Prxs might contribute to almost unaffected RNA expression. Moreover, in *Acanthamoeba*, Prx-1 might be responsible for ROS defense, since we found that only Prx-1 is reduced by Trx [22]. Attempts to establish a qPCR protocol for Prx-1 are still in progress. Treatment with diamide, however, led to increased transcription of Prx-2. Generally, different Prxs might have different functions in the cell and not necessarily all Prxs are induced equally by oxidative stress. In *Candida glabrata* and *S. cerevisiae*, one Prx was described as the main Prx against ROS, while another was proposed to be a backup [16, 31]. Alternatively, Prx-2 and Prx-3 might fulfil other functions, since Prxs were also reported to contribute to genome stability or function as chaperones reviewed by de Oliveira et al. [8]. A more detailed evaluation also on the protein level will be needed to gain greater insights into the metabolism of Prxs in *Acanthamoeba* spp.

In several studies, the high potential of auranofin as an anti-parasitic drug, by inhibiting enzymes involved in the defense against oxidative stress, in particular TrxR, has been demonstrated [7, 9, 12, 37]. In this study, we observed that treatment with auranofin obviously leads to upregulation of enzymes involved in the GSH system, while expression of both TrxRs was at least slightly down-regulated. This might indicate that in *Acanthamoeba*, the Grx/GSH system might act as a backup for the Trx system, not only as an independent redox system but possibly also directly by reducing Trx. This backup option was demonstrated for human Grxs, which are able to reduce Trx1 and Trx2, when TrxR is inhibited [10, 42]. Nonetheless, auranofin exhibited promising antimicrobial activity against *Acanthamoeba* spp. [24]. In *E. histolytica* and *T. vaginalis*, even modest concentrations of auranofin result in increased sensitivity to killing by H_2_O_2_ [9, 19]. Therefore, an investigation of interactions or possible synergistic effects of auranofin with disinfectants or agents currently used in the treatment of AK or GAE would be quite intriguing.

Altogether, our data provide deeper insight into the redox system of *Acanthamoeba* spp. Obviously, both the Trx and the GSH systems are involved in defense against oxidative stress with distinct transcriptional patterns for different stress conditions. While response to stress conditions in different strains generally followed a common pattern, intensity of transcriptional activation varied significantly between strains. Another interesting finding was the divergence in resistance against H_2_O_2_, with the keratitis strain just recently isolated exhibiting extremely high tolerance against H_2_O_2_ as compared to the other strains. Whether resistance against oxidative stress is a common trait in potentially pathogenic isolates remains to be established. However, it appears conceivable since oxidative stress by ROS is part of the defense mechanism of the host’s body. Strategies to impair the oxidative system of *Acanthamoeba* might be a potential way to augment therapy or increase the effect of disinfectant measures, facilitating the prevention of *Acanthamoeba* infections.

## Supplementary Materials

Supplementary material is available at https://www.parasite-journal.org/10.1051/parasite/2022025/olm *Supplementary Table 1*. Fold change (FC) and standard deviation of the mean (SEM) for all investigated target genes for strain Neff after challenge with H_2_O_2_ (H2, H6) and diamide (D2, D6) two and six hours. High-molecular weight thioredoxin reductase (TrxR-L), low-molecular weight thioredoxin reductase (TrxR-S), thioredoxin 1 (Trx-1), peroxiredoxin 2 (Prx-2), glutaredoxin 1 (Grx-1), glutathione reductase (GR), glutathione peroxidase (Gpx), untreated control (C). *Data derived from Leitsch et al. [22]. *Supplementary Table 2*. Fold change (FC) and standard deviation of the mean (SEM) for all investigated target genes for strain Neff after plate culture (NeffPl), three clinical *Acanthamoeba* isolates (2HH, WIR17, JEA19), and strain Neff treated with Auranofin for 48 hours after challenge with H_2_O_2_ (H2) and diamide (D2) for two hours. High-molecular weight thioredoxin reductase (TrxR-L), low-molecular weight thioredoxin reductase (TrxR-S), thioredoxin 1 (Trx-1), peroxiredoxin 2 (Prx-2), glutaredoxin 1 (Grx-1), glutathione reductase (GR), glutathione peroxidase (Gpx), untreated control (C).
