# Supplementary material for: Transcriptional changes of proteins of the thioredoxin and glutathione systems in Acanthamoeba spp. under oxidative stress – an RNA approach
Source: Parasite. 2022 May 9;29:24. doi: 10.1051/parasite/2022025 (PMC9083255; doi:10.1051/parasite/2022025)
Supplement: Supplementary file 1 — Supplementary Table 1. Fold change (FC) and standard deviation of the mean (SEM) for all investigated target genes for strain Neff after challenge with H2O2 (H2, H6) and diamide (D2, D6) two and six hours. High-molecular weight thioredoxin reductase (TrxR-L), low-molecular weight thioredoxin reductase (TrxR-S), thioredoxin 1 (Trx-1), peroxiredoxin 2 (Prx-2), glutaredoxin 1 (Grx-1), glutathione reductase (GR), glutathione peroxidase (Gpx), untreated control (C). *Data derived from Leitsch et al. [22]. [file parasite-29-24-s1.pdf]

Supplementary Table S1. Fold change (FC) and standard deviation of the mean (SEM) for all investigated target genes for strain Neff after challenge with H_2O_2 (H2, H6) and diamide (D2, D6) two and six hours. High-molecular weight thioredoxin reductase (TrxR-L), low-molecular weight thioredoxin reductase (TrxR-S), thioredoxin 1 (Trx-1), peroxiredoxin 2 (Prx-2), glutaredoxin 1 (Grx-1), glutathione reductase (GR), glutathione peroxidase (Gpx), untreated control (C). *Data derived from Leitsch et al. 2021.

Strain	Treat-ment	TrxR-L*		TrxR-S*		Trx-1		Prx-2		Grx-1		GR*		Gpx		Prx-3		Grx-2	
		FC	SEM	FC	SEM	FC	SEM	FC	SEM	FC	SEM	FC	SEM	FC	SEM	FC	SEM	FC	SEM
Neff	C	1.00	0.61	1.00	0.57	1.00	0.42	1.00	0.57	1.00	0.68	1.00	0.32	1.00	0.47	1.00	0.57	1.00	0.47
	H2	0.36	0.14	42.27	14.87	7.31	2.90	1.02	0.25	1.51	0.70	1.73	0.62	1.51	0.44	1.02	0.25	1.51	0.44
	H6	-	-	-	-	4.35	1.54	0.75	0.54	2.34	1.16	-	-	2.50	0.52	1.20	0.13	2.50	0.52
	D2	0.70	0.42	18.08	9.93	13.42	4.55	3.43	1.29	5.76	1.90	5.84	1.73	16.50	5.68	3.43	1.29	16.50	5.68
	D6	-	-	-	-	3.20	1.22	1.03	0.35	2.56	0.98	-	-	4.39	1.57	1.75	0.69	4.39	1.57
