# Supplementary material for: Transcriptional changes of proteins of the thioredoxin and glutathione systems in Acanthamoeba spp. under oxidative stress – an RNA approach
Source: Parasite. 2022 May 9;29:24. doi: 10.1051/parasite/2022025 (PMC9083255; doi:10.1051/parasite/2022025)
Supplement: Supplementary file 2 — Supplementary Table 2. Fold change (FC) and standard deviation of the mean (SEM) for all investigated target genes for strain Neff after plate culture (NeffPl), three clinical Acanthamoeba isolates (2HH, WIR17, JEA19), and strain Neff treated with Auranofin for 48 hours after challenge with H2O2 (H2) and diamide (D2) for two hours. High-molecular weight thioredoxin reductase (TrxR-L), low-molecular weight thioredoxin reductase (TrxR-S), thioredoxin 1 (Trx-1), peroxiredoxin 2 (Prx-2), glutaredoxin 1 (Grx-1), glutathione reductase (GR), glutathione peroxidase (Gpx), untreated control (C). [file parasite-29-24-s2.pdf]

Supplementary Table 2. Fold change (FC) and standard deviation of the mean (SEM) for all investigated target genes for strain Neff after plate culture (NeffPl), three clinical *Acanthamoeba* isolates (2HH, WIR17, JEA19), and strain Neff treated with Auranofin for 48 hours after challenge with H_2O_2 (H2) and diamide (D2) for two hours. High-molecular weight thioredoxin reductase (TrxR-L), low-molecular weight thioredoxin reductase (TrxR-S), thioredoxin 1 (Trx-1), peroxiredoxin 2 (Prx-2), glutaredoxin 1 (Grx-1), glutathione reductase (GR), glutathione peroxidase (Gpx), untreated control (C).

Strain	Treatment	TrxR-L		TrxR-S		Trx-1		Prx-2		Grx-1		GR		Gpx	
		FC	SEM	FC	SEM	FC	SEM	FC	SEM	FC	SEM	FC	SEM	FC	SEM
NeffPL	C	1.00	0.21	1.00	0.43	1.00	0.56	1.00	0.33	1.00	0.27	1.00	0.37	1.00	0.26
	H2	0.52	0.14	97.46	25.75	11.08	3.69	1.81	0.43	2.51	0.65	1.78	0.49	6.65	1.28
	D2	1.35	0.35	32.32	10.79	5.92	1.82	2.05	0.53	2.26	0.52	2.15	0.55	11.96	1.82
WIR17	C	1.00	0.33	1.00	0.35	1.00	0.26	1.00	0.37	1.00	0.38	1.00	0.26	1.00	0.26
	H2	0.33	0.09	74.66	22.97	4.54	0.95	1.38	0.38	1.03	0.22	0.71	0.18	2.05	0.43
	D2	0.68	0.18	18.66	4.88	6.14	2.22	2.77	0.74	2.43	0.70	2.46	0.57	7.51	2.77
2HH	C	1.00	0.37	1.00	0.55	1.00	0.39	1.00	0.41	1.00	0.08	1.00	0.29	1.00	0.33
	H2	0.12	0.06	11.33	4.85	6.21	1.71	0.84	0.23	1.67	0.44	0.73	0.33	1.66	0.49
	D2	0.28	0.13	14.45	6.38	17.37	5.90	3.24	1.10	4.84	1.60	4.57	1.57	31.02	9.98
JEA19	C	1.00	0.42	1.00	0.37	1.00	0.30	1.00	0.30	1.00	0.27	1.00	0.40	1.00	0.33
	H2	0.29	0.10	15.33	6.58	4.84	1.21	1.00	0.17	0.70	0.16	1.04	0.33	2.02	0.42
	D2	0.62	0.25	6.71	3.55	8.10	2.17	2.39	0.43	1.53	0.36	2.67	0.89	7.31	2.78
Neff Auranofin	C	1.00	0.19	1.00	0.28	1.00	0.40	1.00	0.21	1.00	0.32	1.00	0.27	1.00	0.26
	H2	0.92	0.24	0.81	0.29	1.79	0.35	1.27	0.36	1.84	0.23	1.77	0.26	1.91	0.31
	D2	0.68	0.14	0.66	0.23	2.83	0.23	1.17	0.33	2.61	0.16	2.63	0.29	2.67	0.26
